# Experimental investigation of borehole blasting with eco friendly rapid setting gun mud

**DOI:** 10.1038/s41598-025-19060-0

**Published:** 2025-10-08

**Authors:** Hou-You Zhou, Xiao Wang, Zheng-Hua Gao, Xin Zhao, En-An Chi, Wen-Bo Zhao

**Affiliations:** 1 School of Mechanics and Civil Engineering, China University of Mining and Technology-Beijing, Beijing, 100083 China; 2 School of Materials and Achitectural Engineering (Guizhou School of Emergency Management), Guizhou Normal University, Guiyang, 550025 China; 3 China Railway 19th Bureau Group Pilot Technology Corrorationg Limited, Beijing, 100176 China; 4 Poly Union Group Co., Ltd, Guiyang, 550002 China

**Keywords:** Drilling and blasting, Borehole sealing, Borehole sealing materials, Tunnel blasting

## Abstract

To address the challenges of inconvenient material sourcing and low stemming efficiency in horizontal tunnel blasting, this study proposes a novel rapid-setting gun mud. The material’s composition, formation mechanism, and initial setting characteristics are first analyzed, and a multi-parameter coupled model for optimal stemming length is established. Subsequently, a series of blasting model tests with varying stemming lengths is conducted, followed by a quantitative analysis of the block-size distribution of limestone under different stemming conditions. The influence of stemming length on rock fragmentation characteristics is revealed, and theoretical predictions are validated against experimental results. Findings indicate that the curing process of the proposed material is primarily driven by hydration reactions, with simple operation, environmental friendliness, and safety for workers. Moreover, the developed multi-parameter coupled model for optimal stemming length shows good agreement with experimental results, providing a quantifiable theoretical basis for the refined parameter design in mechanized tunnel blasting.

## Introduction

Blasting operations offer simple implementation, strong economic return and rapid progress, and have been broadly applied in transportation infrastructure, municipal works, hydraulic projects and mineral extraction^1–5^. In tunnel engineering, particularly in mountainous‐region highway tunnels, drill‐and‐blast remains the principal excavation and advance method^6–13^. However, extensive practice has shown that the energy utilization of explosive charges in open drill holes is very low; consequently, effective borehole sealing measures are normally required to enhance blasting performance^14–16^. Because sealing not only affects blasting efficiency but also influences ore recovery and dilution rates in mining, it is essential to quantify how sealing conditions impact rock fragmentation^17,18^.

In current practice, borehole sealing is neither consistently applied nor always appropriate. In most upward or horizontal drilled blasts, either no sealing is employed or only small plastic plugs or similar materials are used^19,20^. Field evidence demonstrates that, without proper sealing, up to 50% of the explosive energy may escape directly through the borehole mouth^21^. Conversely, effective stemming can increase the explosive’s destructive power by at least 41%, and even simple seals can boost energy utilization by some 60%^22^; well‐designed sealing measures may raise it by as much as 93%^23^. Moreover, inadequate borehole sealing not only degrades fragment size distribution but also exacerbates environmental impacts—ground vibration, noise, air shock waves and flying stone^24^.

Three categories of sealing materials are commonly used in blasting: colloidal, liquid and solid^25–30^. To systematically investigate how borehole sealing affects rock breakage, the most effective approaches combine model‐scale tests, in situ trials and numerical simulation^31–35^. For example, small‐scale model tests have shown that different sealants perform various functions and significantly influence rock‐breaking efficiency^36^. Other researchers have developed an initiation material based on a shear‐thickening fluid (STF) responsive to dynamic pressure^37^. Two blast experiments, evaluated via image‐processing of fragment sizes, demonstrated the STF‐based material’s superior sealing performance. Li et al.^38^ applied a water‐filled polyethylene bag technique with good results, while another study proposed a water‐sand composite seal for tunnels to enhance fragmentation, reduce dust and lower explosive consumption^39^. Zhu et al.^40^ performed AUTODYN simulations under dynamic loading using various sealants (water, sand, air) and achieved notable rock‐breaking improvements.

We have conducted a comprehensive analysis of both novel and traditional borehole fillers. STF, though effective at buffering shock waves and limiting vibration damage, entails complex preparation, solvent evaporation and phase separation, and high equipment and raw‐material costs, making it suitable only for small‐volume, high‐precision blasts. Polyethylene water bags are inexpensive but require separate filling and risk rupture from borehole wall friction. Traditional fillers such as clay, drill cuttings and gravel are mainly used in vertical boreholes at open‑pit mines; their use in horizontal holes is labor‐intensive, inconvenient to source and ill-suited to the rapid, efficient requirements of tunnel blasting, especially in ordinary highway and railway tunnels (see Table 1).

**Table 1:** The main ingredients of quick-setting gun clay material.

Material	Advantages and applicable Scenarios	Disadvantages
STF	Effectively cushions shock waves and reduces blasting vibrations, making it suitable for high-precision applications such as urban demolition	Requires multiple steps and specialized equipment; reagents and tools are costly
PE‑lined water bags	Very low cost; ideal for blasting operations that also require dust suppression	Needs on-site water filling; bags can tear from borehole friction, causing leaks
Traditional fillers (clay, drill cuttings, gravel, etc.)	Cheap and easy to obtain; commonly used in large-diameter vertical boreholes in open-pit mining	Poor fit for horizontal boreholes: installation is labor-intensive, time-consuming, and suitable fill is often hard to find locally

In practice, incomplete or omitted sealing—due to technological, cost or efficiency constraints—has become commonplace in tunnel blasts, significantly impairing excavation effectiveness and increasing blasting hazards^41–44^. Therefore, it is imperative to develop a rapid, efficient borehole filler tailored to horizontal tunnel applications. Rapid‐setting materials, with excellent fluidity and fast solidification, are receiving growing attention: they quickly form high‐strength seals, are simple to prepare and can be rapidly injected into horizontal holes to meet diverse blasting needs.

Against this backdrop, the present study examines a novel rapid‐setting gun mud. We first analyze its composition, setting mechanism and initial‐setting characteristics, then propose a multi‐parameter coupled model for its optimal stemming length. Combining theoretical derivation with drill‐and‐blast model tests, we quantitatively evaluate the sealing performance of the rapid‐setting mud and elucidate how variations in stemming length affect rock‐fragmentation characteristics, thereby providing a quantifiable theoretical foundation for precise sealing‐parameter matching in intelligent tunnel‐blasting design.

## Key components and initial setting characteristics of the rapid-setting borehole sealing material

### Primary components

The rapid-setting borehole sealing material employed in this study is developed through modifications of high-water-content materials and comprises two composite components, designated as Component A and Component B. Component A is primarily composed of sulfoaluminate cement clinker, bentonite, set retarders, and dispersants, while Component B mainly contains natural gypsum, quicklime, calcium carbonate, accelerators, and early-strength agents. The principal constituents are summarized in Table 2.

**Table 2:** The main ingredients of quick-setting gun clay material.

Component	Main constituents
Material A	80.5–94.1% sulfoaluminate cement clinker, 4.7–14.9% bentonite, 0.27–3% set retarders, 0.09–2.1% dispersants
Material B	36–69% natural gypsum, 4–19% quicklime, 20–51% calcium carbonate, 2–5% accelerators, 0.1–1% early-strength agents

**Fig. 1:**
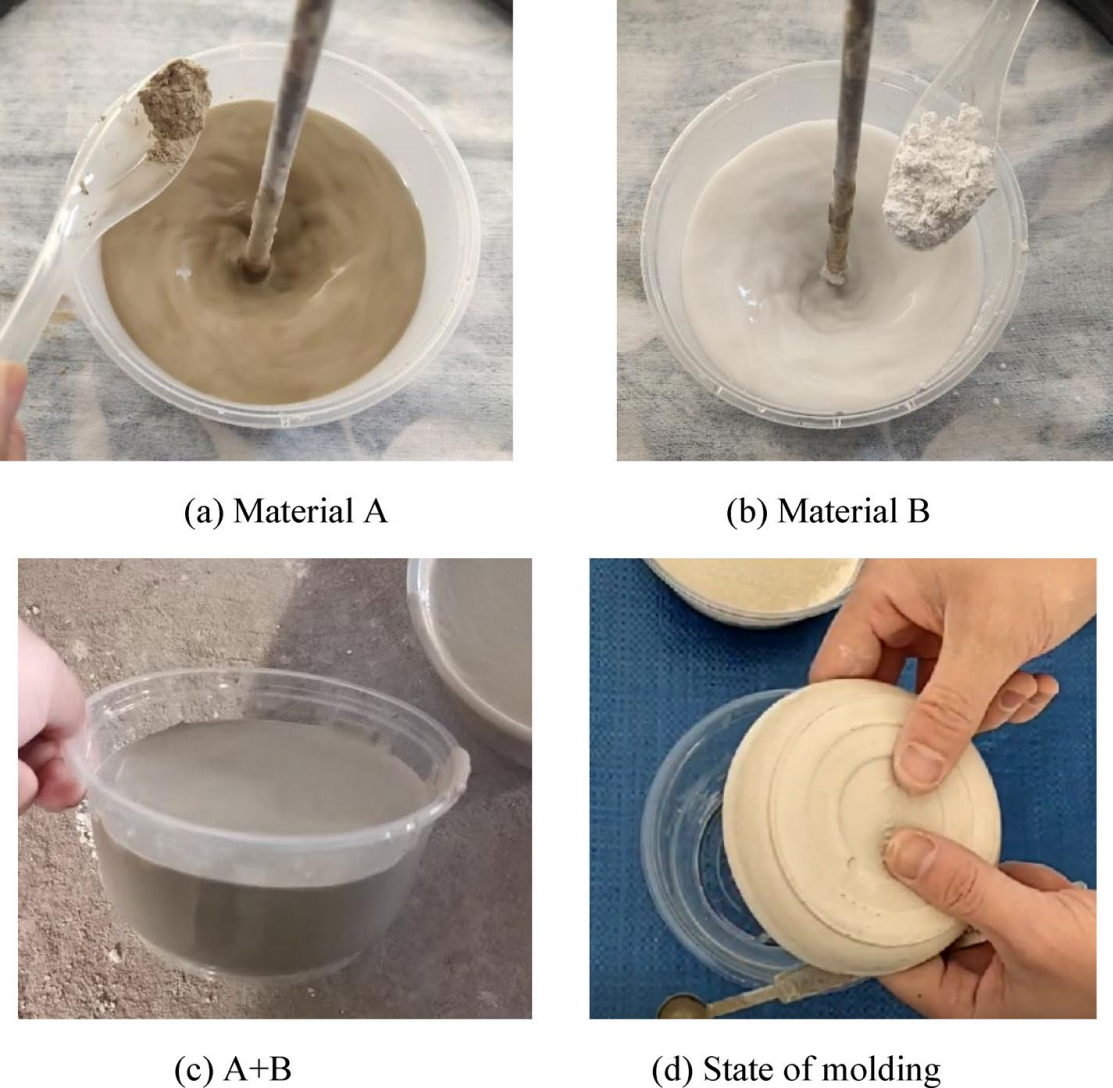
The solidification process of quick-setting gun mud materials.

As illustrated in Fig. 1, Components A and B are each first blended with water at a mass ratio of 1:1 to yield two separate slurries. These slurries are then combined in a 1:1 volume ratio and immediately undergo a hydration reaction, forming a rapid‑setting gun‑mud slurry that swiftly solidifies into a cohesive plug. Once mixed, the composite slurry can be conveyed to the borehole via a mechanical pumping system and piping network, achieving initial set within just a few seconds. The initial setting time ranges from approximately 30 s to 300 s, and the material exhibits an exceptionally high retention rate within the borehole.

The dominant hydration product of the high-water-content material is ettringite; hence, the key to forming the rapid-setting borehole sealing material lies in the rapid and abundant formation of ettringite in the mixed slurry. The hydration reactions of both the primary and secondary components, which lead to the formation of a consolidated structure, are complex. In practice, the two components are prepared as separate slurries and mixed only upon injection into the borehole, where the subsequent reaction produces the consolidated sealing material. Analysis of the material composition reveals that the main reactive species are ${\text{C}}_{4}{\text{A}}_{3}\overline{\text{S}}$, β-C2S, polymorphs, calcium sulfate, and lime). Their hydration processes and the resulting products determine the principal mechanical properties of the consolidated body. Three key reaction pathways are observed:

1. Complete Ettringite Formation: When sufficient reactants are present, the entire system hydrates to form ettringite.
  1 $$C_{4} A_{3} \overline{S} + 8\left( {CaSO_{4} \cdot 2H_{2} O} \right) + 6CaO + 96H_{2} O \to 3\left( {3CaO \cdot Al_{2} O_{3} \cdot 3CaSO_{4} \cdot 32H_{2} O} \right)$$
2. Partial Formation with Aluminum Gel: When the lime content is insufficient, not all products are ettringite; instead, a portion of aluminum gel is formed.
  2 $$C_{4} A_{3} \overline{S} + 2\left( {CaSO_{4} \cdot 2H_{2} O} \right) + 34H_{2} O \to 3CaO \cdot Al_{2} O_{3} \cdot 3CaSO_{4} \cdot 32H_{2} O + 2\left( {Al_{2} O_{3} \cdot 3H_{2} O} \right)$$
3. Formation of AFm and Aluminum Gel: When the sulfate content is inadequate, ettringite no longer precipitates; instead, AFm phases and aluminum gel form, and previously formed ettringite may decompose into AFm.
  3 $$C_{4} A_{3} \overline{S} + 18H_{2} O \to 3CaO \cdot Al_{2} O_{3} \cdot CaSO_{4} \cdot 12H_{2} O + 2\left( {Al_{2} O_{3} \cdot 3H_{2} O} \right)$$
  4 $$\begin{aligned} & 3CaO \cdot Al_{2} O_{3} \cdot 3CaSO_{4} \cdot 2H_{2} O \to \\ & 3CaO \cdot Al_{2} O_{3} \cdot CaSO_{4} \cdot 12H_{2} O + 2\left( {CaSO_{4} \cdot 2H_{2} O} \right) + 16H_{2} O \\ \end{aligned}$$

The addition of calcium carbonate in the formulation partly inhibits the formation of AFm, thereby improving the setting time of the sealing material. Furthermore, another major component of the sulfoaluminate cement, β-C₂S, hydrates to form calcium silicate hydrate (C-S–H) gel. The calcium hydroxide released during this process subsequently reacts with the aluminum gel and calcium sulfate to form additional ettringite, as represented by the reaction equation below:

5 $$\beta - {\text{C}}_{2} {\text{S}} + 2{\text{H}}_{2} {\text{O}} \to {\text{C}} - {\text{S}} - {\text{H}} + {\text{Ca}}\left( {{\text{OH}}} \right)_{2}$$

6 $$3Ca\left( {OH} \right)_{2} + 3\left( {CaSO_{4} \cdot 2H_{2} O} \right) + Al_{2} O_{3} \cdot 3H_{2} O \to 3CaO \cdot Al_{2} O_{3} \cdot 3CaSO_{4} \cdot 32H_{2} O$$

By promoting the hydration of β-C₂S and accelerating the formation of C-S–H, the pores between the structural skeleton formed by ettringite are effectively filled and bonded, rendering the consolidated structure denser. Although the overall hydration process is subject to diffusion limitations and cannot be described by a single, precise chemical equation, the above equations provide a theoretical framework to explain the formation of ettringite in the rapid-setting sealing material. This explanation aids in understanding the formation mechanism; detailed mechanistic intricacies are beyond the scope of this article.

### Initial set characteristics

**Fig. 2:**
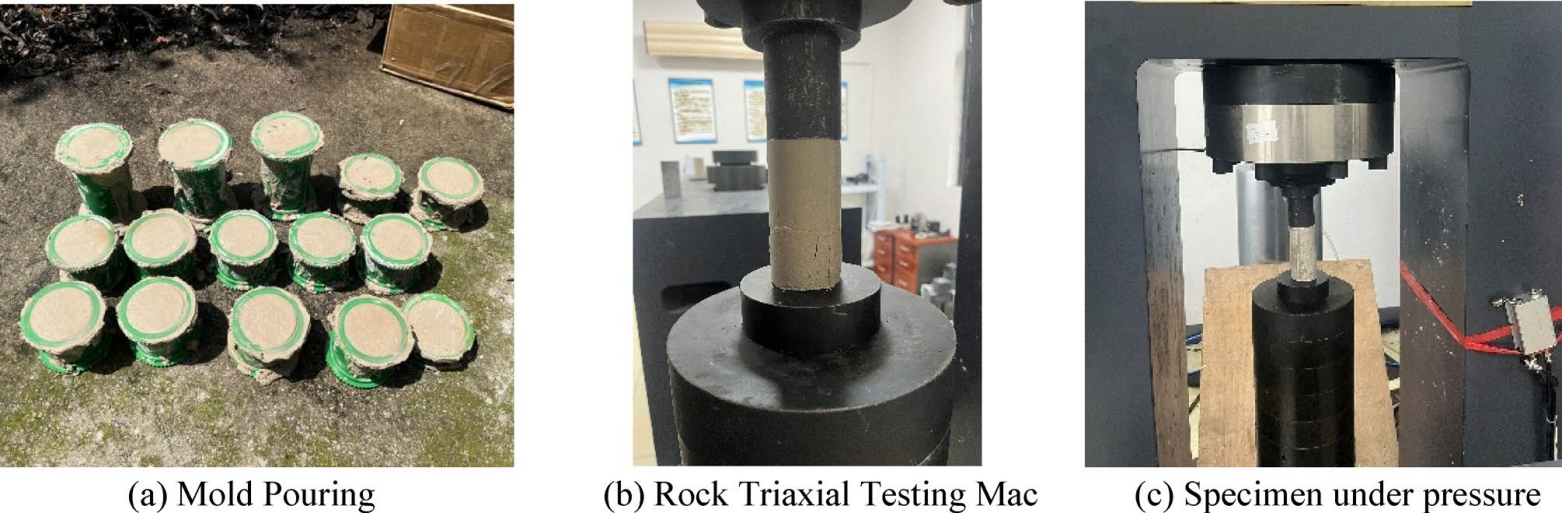
Uniaxial compression test.

According to the mix proportions in Table 2, the rapid‑setting gun‑mud was cast into standard specimens of φ50 mm × 100 mm. At the Poly Union Group Co. , Ltd , Civil Explosives Laboratory, a TAJW‑2000 microcomputer‑controlled electro‑hydraulic servo rock triaxial testing machine was employed to measure the strength evolution of the gun‑mud at 15 min, 30 min, 45 min, and 60 min after initial set. The experimental setup and procedure are shown in Fig. 2.

**Fig. 3:**
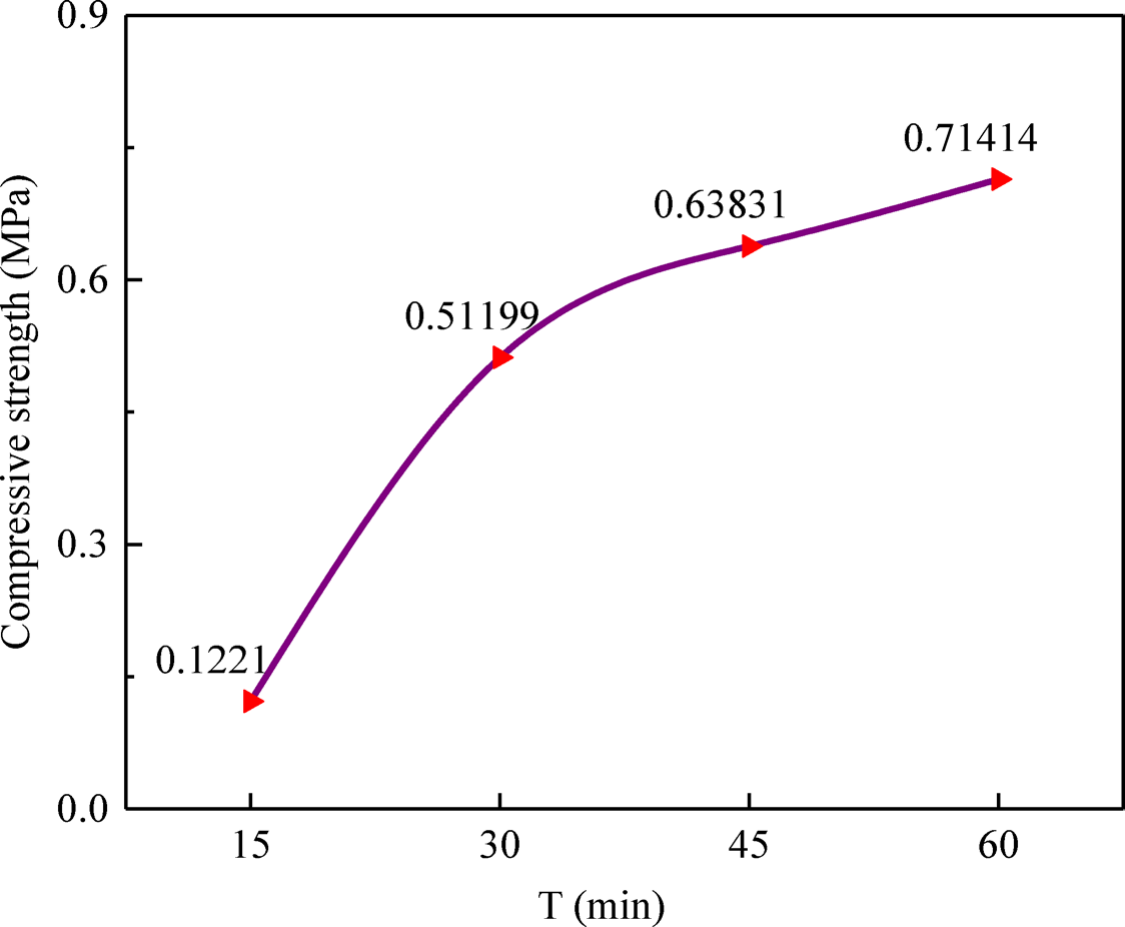
Strength change of quick-setting gunite within 1 h after initial setting.

Each test was conducted in triplicate, and the average compressive strength was recorded. Prior to testing, the contact surfaces of the loading platens were meticulously cleaned to ensure they were free of dust. The loading was applied uniformly at a rate of 0.005 kN/s until an audible fracture was detected and an inflection point appeared on the load curve (see Fig. 3).

The results indicate that the compressive strength of the rapid-setting sealing material increases continuously with time after initial set, though the rate of strength gain gradually diminishes. Specifically, the strength at 15 min after initial set is 17.10% of the 1-h strength, at 30 min it reaches 71.69%, and at 45 min it attains 89.38% of the 1-h strength. These results demonstrate a pronounced early strength development within the first 30 min, followed by a plateau.

**Fig. 4:**
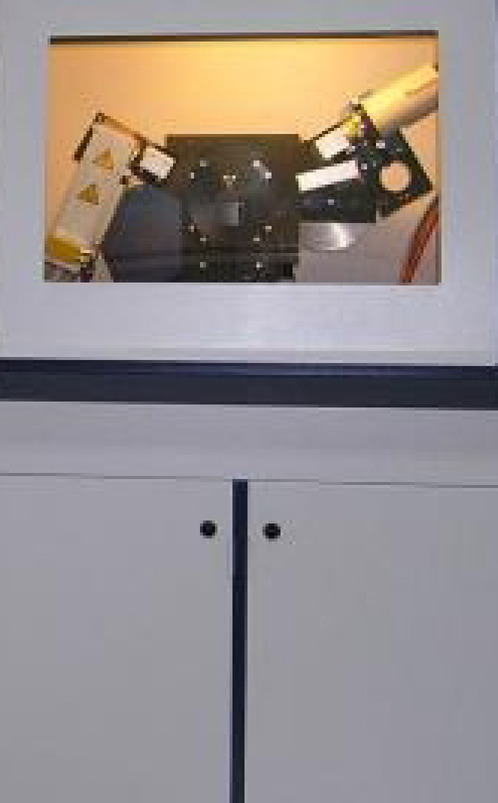
X-ray diffractometer.

**Fig. 5:**
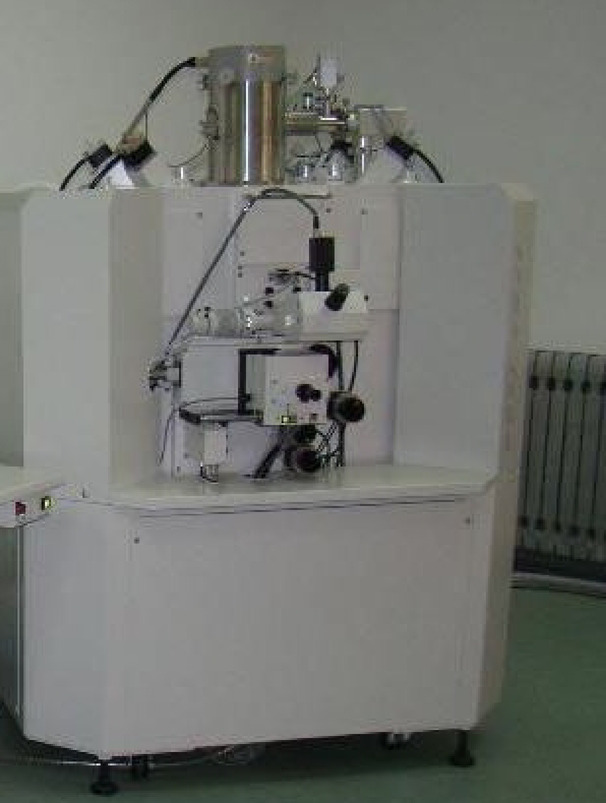
Scanning electron microscopy.

**Fig. 6:**
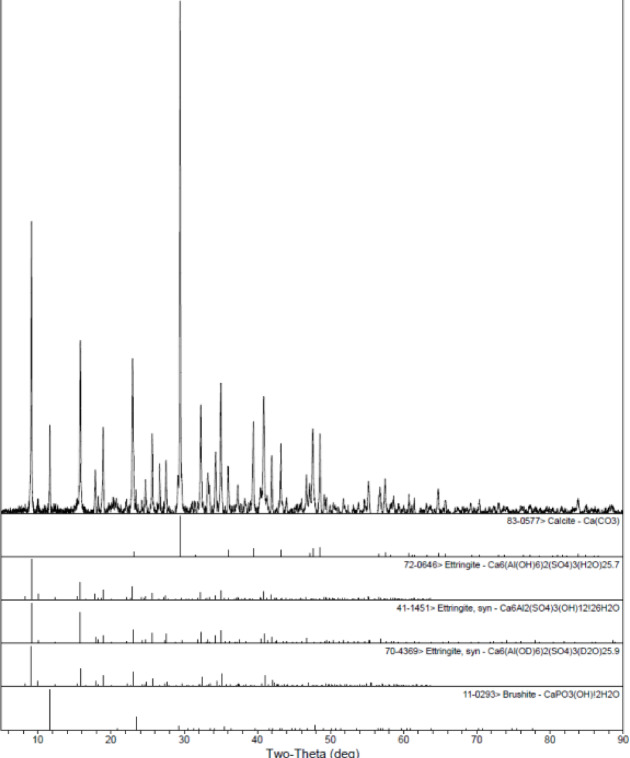
XRD test results.

Additionally, to further elucidate the hydration products, the consolidated body formed 30 min after initial set was analyzed by X-ray diffraction (XRD) and scanning electron microscopy (SEM), as shown in Figs. 4 and 5. XRD analysis reveals that the primary phases present are ettringite and its polymorphs, with additional phases of calcium carbonate and tricalcium phosphate detected. The calcium carbonate likely originates from the unreacted fraction of Component B, while the presence of tricalcium phosphate is attributed to impurities in the raw materials, though its precise source remains unclear. Notably, no low-sulfur AFm phases were detected, supporting the hypothesis that the addition of calcium carbonate effectively inhibits the transformation of high-sulfur phases to low-sulfur AFm. Since the calcium silicate hydrate (C-S–H) gel is amorphous, it is not detectable by XRD (see Fig. 6).

**Fig. 7:**
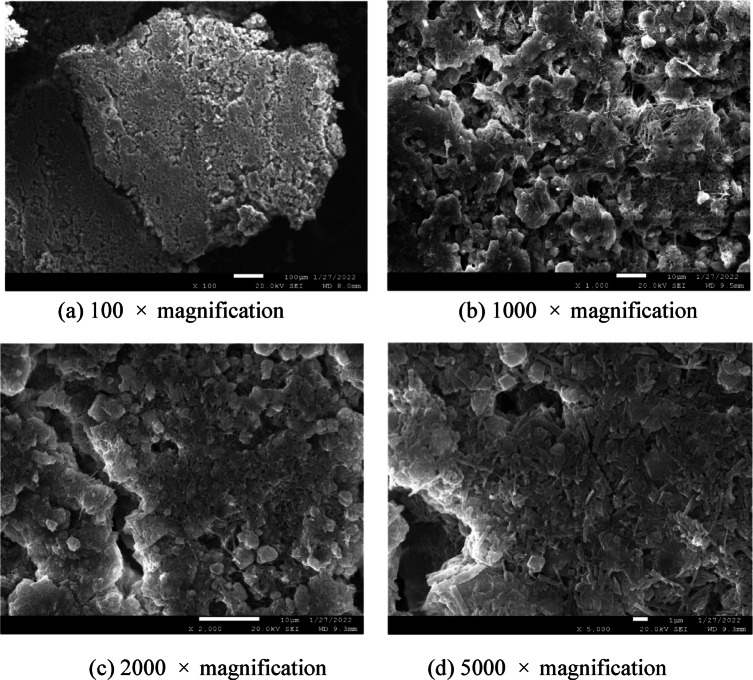
Scanning electron microscope test results.

SEM observations (Fig. 7) reveal that the microstructure of the rapid-setting sealing material consists predominantly of two structural forms: a fibrous network and a layered, plate-like structure. These two interpenetrating and interconnecting structures provide a skeletal framework that reinforces the consolidated body. The extensive contact area between the crystals facilitates effective coupling with the borehole walls, thereby enhancing the quality of the borehole filling.

In summary, a concise analysis of the rapid‑setting gun‑mud’s primary composition and formation mechanism reveals that it is a high‑water‑content material produced principally by the hydration of water, cement, gypsum, calcium carbonate, and accelerator, releasing no toxic by‑products and thus posing no adverse effects to operators or the surrounding environment. Moreover, strength testing demonstrates that the material achieves initial set within seconds (approximately 30–300 s) and develops a stable strength in about 30 min thereafter, ensuring a rapid transition from a fluid slurry to a self‑supporting solid plug upon borehole injection—fully satisfying both the practical demands of the research and its application value.

## Multi-parameter coupled optimal stemming length model

### Fundamental assumptions and kinematic analysis

According to the rock fragmentation mechanism of blasting, explosives exhibit extremely rapid detonation velocities, and the detonation of cylindrical charges is completed within a very short time, during which a crushed zone is formed and cracks initiate in the surrounding rock mass. Consequently, the loading process of the stemming structure during blasting is highly complex. To facilitate engineering applications, numerous researchers have proposed various simplified models and assumptions in analyzing the kinematics of borehole stemming materials, which have yielded satisfactory results and provided valuable insights for studying the dynamics and optimal length design of stemming materials. For instance, Reference^44^ assumes that the stemming structure undergoes uniformly accelerated motion over a short time interval, and that the gas-driven motion of the stemming along the borehole axis can be treated as one-dimensional. Reference^45^ models the stemming as a rigid body and considers its motion as rigid body translation. Reference^46^ assumes that the stemming undergoes uniform linear acceleration within the borehole, treats the detonation gases as ideal gases, and considers the compressed stemming as a rigid body. In line with these approaches and for the sake of analyzing the stemming motion in the borehole, the present study adopts the following assumptions:

1. The explosive detonation is instantaneous, allowing the expansion of the explosive gases to be treated as one-dimensional^44^.
2. The explosive gases behave as an ideal gas^45^.
3. The movement of the stemming material is assumed to be that of a rigid body (i.e., it is incompressible) undergoing uniform acceleration^45,46^.

**Fig. 8:**
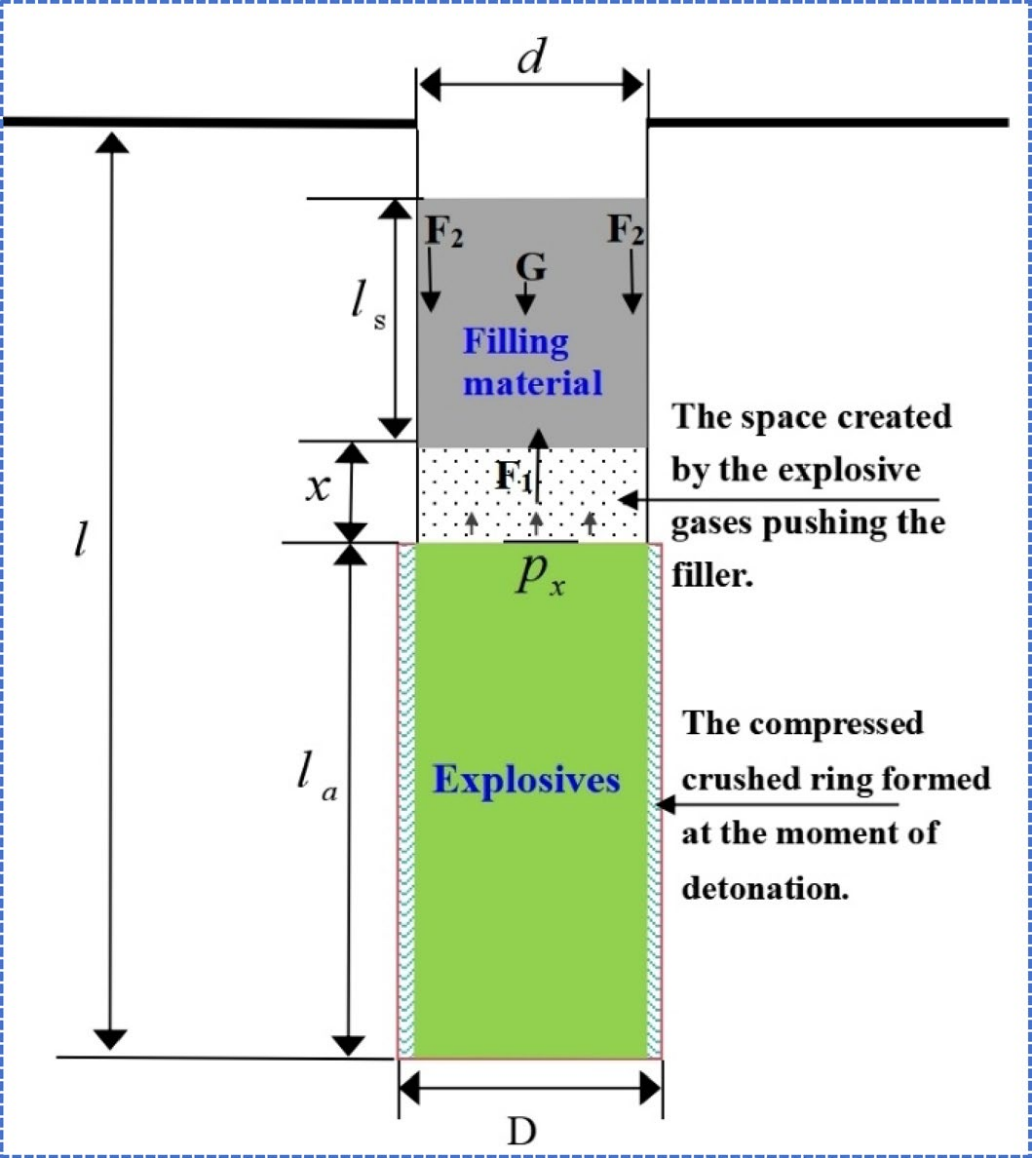
Mechanics model of the stem in borehole.

Based on these assumptions and the theoretical foundations of rock blasting mechanics, the kinematic model for the rapid-setting sealing material in the borehole is depicted in Fig. 8. Its motion conforms to Newton’s second law, whereby the net force on the plug is expressed as follows:

7 $$F = M \cdot a$$

8 $$F = F_{1} - F_{2} - G$$

where:

9 $$F_{1} = \frac{{\pi d^{2} p_{x} }}{4}$$

10 $$M = \pi r^{2} l_{s} \rho_{0} = \frac{{\pi d^{2} l_{s} \rho_{0} }}{4}$$

In the equation, where $F$ is the resultant force acting on the stemming material (N), $a$ is the acceleration of the stemming material (m/s^2^), and M is its mass (kg). $F_{1}$ denotes the thrust exerted by the explosion-generated expanding gas toward the outside of the borehole (N), while $F_{2}$ represents the frictional force between the stemming material and the borehole wall (N). G is the self-weight of the stemming material (N). $d$ denotes the diameter of the borehole prior to detonation (m), $p_{x}$ is the gas pressure inside the borehole after the explosive detonation (Pa), $l_{s}$ is the length of the stemming column (m), and $\rho_{0}$ is the density of the stemming material (kg/m^3^).

Under the static pressure of the explosive gases, an axial compressive stress develops within the stemming material, equal to $p_{x}$. Due to the axial loading, the material tends to expand radially; however, the constraint imposed by the borehole wall restricts this expansion, thus generating frictional resistance. According to the Poisson effect (see Fig. 8), the frictional force $F_{2}$ encountered by the plug during its movement can be calculated by the following equation:

11 $$F_{2} = \pi d{\text{s}}\lambda fp{}_{x}$$

12 $$\lambda = \frac{{\mu_{{\text{d}}} }}{{1 - \mu_{{\text{d}}} }}$$

where S is the contact length between the stemming material and the borehole wall, $f$ is the coefficient of sliding friction, $\mu_{d}$ sigma is the dynamic Poisson’s ratio of the rock (typically 0.8 times its static Poisson’s ratio under blasting loading conditions). From Eqs. (8)–(12), the acceleration of the stemming material can be derived.

13 $$F = \left( {\frac{{\pi d^{2} l_{s} \rho_{0} }}{4}} \right)a = \frac{{\pi d^{2} p_{x} }}{4} - \pi ds\lambda fp{}_{x} - G$$

14 $$a = \left( {\frac{{d - 4{\text{s}}\lambda f}}{{dl_{{\text{s}}} \rho_{0} }}} \right)p{}_{x} - {\text{g}}$$

Since the contribution of gravitational acceleration to other forces can be neglected, it can be omitted in engineering calculations, thereby simplifying the expression to:

15 $$a = \left( {\frac{{d - 4{\text{s}}\lambda f}}{{dl_{{\text{s}}} \rho_{0} }}} \right)p{}_{x}$$

Further analysis of the rock blasting mechanism reveals that once the crushed zone forms, the explosive gases rapidly fill the borehole, pushing the stemming material to move as a whole. During this phase, the gas pressure in the borehole is nearly static and can be approximated by the ideal gas law. Although this approximation deviates somewhat from reality, it is sufficient for engineering computations. The state equation for the explosive gases is given by^45^:

16 $$pV^{\gamma } = const$$

where $p$ is the gas pressure (Pa), V is the gas volume (m^3^), and γ is the adiabatic index (typically 3 for industrial explosives).

According to the assumptions illustrated in Fig. 8, the volume expansion work done by the explosive gases originates from two sources: 1) the displacement of the stemming material increases the volume, and 2) the formation of microfractures in the borehole wall. However, the latter is negligible, leading to the derivation of Eq. (16):

17 $$p_{x} = \left( {\frac{{V{}_{1}}}{{V_{1} + \frac{{\pi d^{2} x}}{4}}}} \right)^{3} p_{1}$$

18 $$p_{1} = \left( {\frac{{V{}_{0}}}{{V_{1} }}} \right)^{3} p_{0}$$

19 $$V_{0} = \frac{{\pi d^{2} l_{a} }}{4}$$

20 $$V_{1} = \frac{{\pi D^{2} l_{a} }}{4}$$

where: V_0_ is the borehole volume of the stemming section before detonation (m^3^), $V_{x}$ is the borehole volume of the stemming section after the formation of the crushed zone (m^3^), The initial pressure exerted by the explosive gases on the borehole wall is $p_{0}$ (Pa), D is the diameter of the crushed zone (m), $x$ is the distance the stemming material is pushed along the borehole by the gas expansion ($x = l - l_{s} - l_{a}$_,_m), and $l_{a}$ is the length of the charge (m).

Combining Eqs. (17)–(20) yields:

21 $$p_{x} = \left( {\frac{{\frac{{\pi D^{2} l_{a} }}{4}}}{{\frac{{\pi D^{2} l_{a} }}{4} + \frac{{\pi d^{2} x}}{4}}}} \right)^{3} \left( {\frac{{\frac{{\pi d^{2} l_{a} }}{4}}}{{\frac{{\pi D^{2} l_{a} }}{4}}}} \right)^{3} p_{0}$$

22 $$p_{x} = \left( {\frac{1}{{\left( \frac{D}{d} \right)^{2} + \frac{x}{{l_{a} }}}}} \right)^{3} p_{0}$$

Which upon simplification (after defining the ratio of the crushed zone diameter to the borehole diameter as $\xi$) ,

Literature indicates that, in engineering practice, the extent of the crushed zone rarely exceeds 3–5 times the borehole radius^47^. For engineering convenience, a factor of 4 is adopted, i.e., $\xi$ = 4. In typical tunnel drilling, additional minor parameters can be neglected due to their negligible magnitudes. Furthermore, considering the practical conditions of tunnel borehole drilling—where typically $x$ is less than $l_{a}$ (i.e., $0 \le \frac{x}{{l_{a} }} < 1$) and $\frac{x}{{l_{a} }}$ is nearly zero compared to the preceding term—the value of $\frac{x}{{l_{a} }}$ can be neglected in engineering calculations for simplicity.

Moreover, under axially continuous charge conditions, the initial pressure of the explosive gases filling the borehole can be calculated by the following equation^45,48^:

23 $$p_{0} = \frac{{\rho_{1} D_{c}^{2} }}{2(\gamma + 1)}$$

where $\rho_{1}$ is the explosive density (kg/m^3^), $D_{c}$ is the detonation velocity (m/s), and $\gamma$ is the adiabatic index (taken as 3 for industrial explosives).

### Optimal stemming length model analysis

**Fig. 9:**
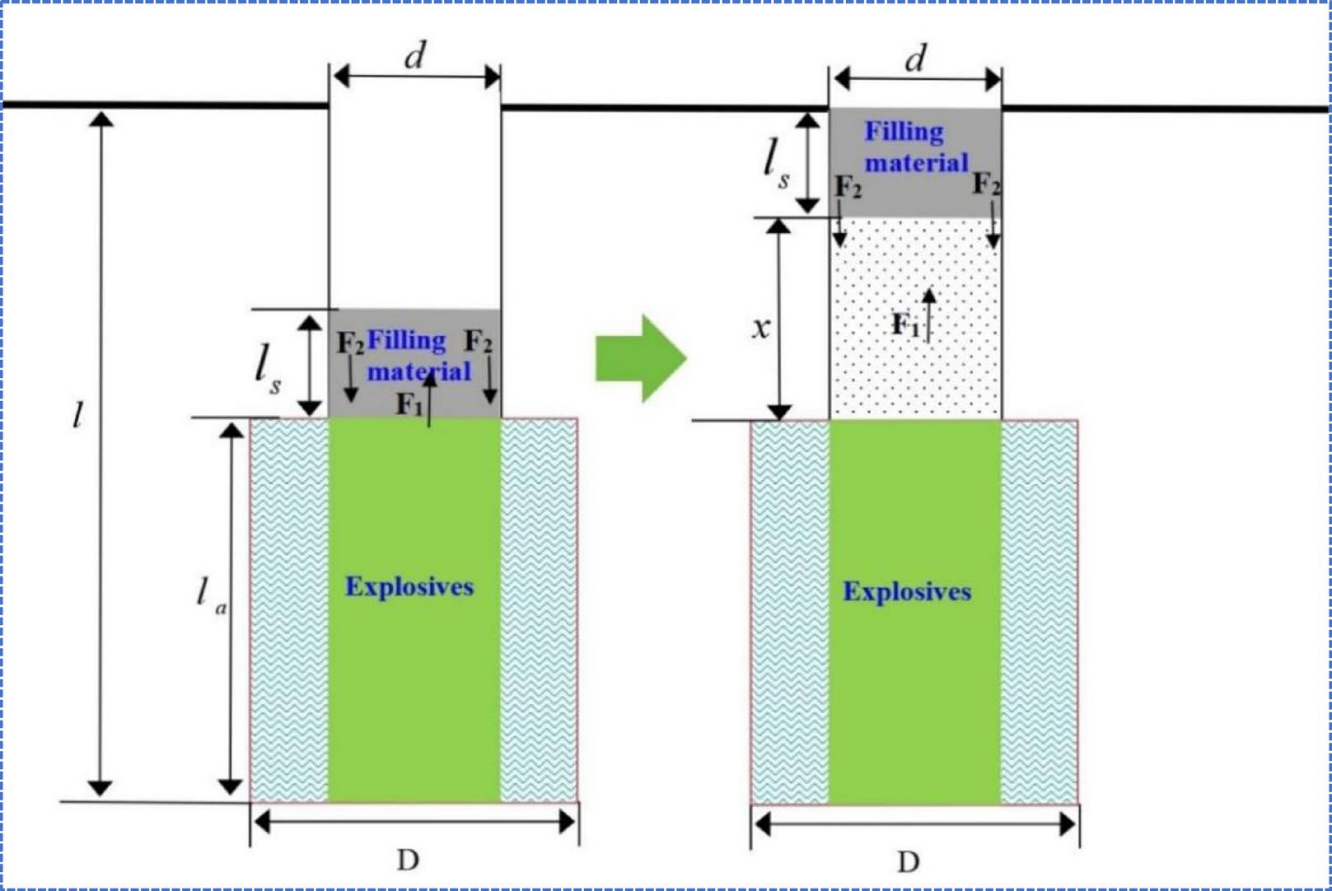
Improved optimal length kinematic model for shell hole fillers.

The optimization of stemming length is of great significance for improving blasting efficiency and reducing stemming costs. In this study, based on practical field blasting conditions, we propose that the optimal stemming length should ensure that the stemming material remains within the borehole during the rock-fracturing period, with its top just reaching the borehole collar at the end of this period. As illustrated in Fig. 9, when the stemming length reaches its optimal value, the top of the stemming material arrives precisely at the borehole mouth at the end of the rock-fracturing time $t_{p}$.

Under the assumption that the plug does not compress during movement, the contact length between the plug and the borehole remains constant and is equal to the stemming length, i.e., $S = l_{s}$. Thus, Eq. (15) can be rewritten accordingly.

24 $$a= Px (d - 4l_{s} \lambda f) /(dl_{s} \rho_{0}) $$

Furthermore, the effective time of the stemming material in the borehole comprises both the compression time due to the shock wave and the plug’s displacement time. However, because the shock wave propagates extremely fast, its compression time is negligible. Therefore, the plug’s movement time is approximately equal to the time required for the rock to fracture into fragments, and t_p_ can be calculated using the following formula:

25 $$t_{p} = \frac{2w}{{C_{p} }} + \frac{w}{{C_{R} }}$$

where $w$ is the minimum resistance line (or critical dimension), and $C_{P}$ and $C_{R}$ are the longitudinal and Rayleigh wave velocities in the rock, respectively. Using the equations of uniformly accelerated motion ($x = \frac{{at_{p}^{2} }}{2}$), we obtain:

26 $$x = \frac{{p_{x} t_{p}^{2} }}{2}\left( {\frac{{d - 4l_{s} \lambda f}}{{dl_{s} \rho_{0} }}} \right)$$

And $$x = l - l_{s} - l_{a}$$:

27 $$l - l_{s} - l_{a} = \frac{{p_{x} t_{p}^{2} d - p_{x} t_{p}^{2} 4l_{s} \lambda f}}{{2dl_{s} \rho_{0} }}$$

So:

28 $$l_{s}^{2} (d\rho_{0} ) + l_{s} (l_{a} d\rho_{0} - 2p_{x} t_{p}^{2} \lambda f - ld\rho_{0} ) + \frac{{p_{x} t_{p}^{2} d}}{2} = 0$$

Equation (28) is a univariate quadratic equation, let

29 $$A = (d\rho_{0} )$$

30 $$B = (l_{a} d\rho_{0} - 2p_{x} t_{p}^{2} \lambda f - ld\rho_{0} )$$

31 $$C = \frac{{p_{x} t_{p}^{2} d}}{2}$$

Solving Eq. (28) yields:

32 $$l_{s} = \frac{{ - B \pm \sqrt {B^{2} - 4AC} }}{2A}$$

In practical engineering, it is recognized that the actual energy imparted to the plug is lower than the theoretical value due to factors such as pre-existing fractures in the rock and local damage at the borehole mouth, which lead to significant energy losses, particularly in the gas pressure. To account for these discrepancies, a correction coefficient is applied to the model ($k$). The final optimal stemming length model thus considers multiple parameters, reflecting the complexity of factors influencing the optimal length.

33 $$l_{s} = \frac{{(l_{a} d\rho_{0} - 2p_{x} t_{p}^{2} \lambda f - ld\rho_{0} ) \pm \sqrt {(l_{a} d\rho_{0} - 2p_{x} t_{p}^{2} \lambda f - ld\rho_{0} )^{2} - 2d\rho_{0} p_{x} t_{p}^{2} d} }}{{2kd\rho_{0} }}$$

The final optimal stemming length model thus considers multiple parameters, reflecting the complexity of factors influencing the optimal length. And $k$ typically ranging from 1.1 to 1.5, adjustable according to the degree of rock fracturing and surrounding rock conditions. Under specified blasting conditions, where the parameters for the rock, explosive, drilling, and sealing material are known, Eqs. (22)–(33) can be used to compute the optimal stemming length for axially continuous charges. The “ ± ” in Eq. (33) requires further experimental verification.

## Physical model blasting tests

### Model design and fabrication

At present, cast concrete specimens with comparable mechanical strength are the most widely used materials in blasting model tests due to their low cost, ease of fabrication, and high efficiency. However, when conditions permit, in-situ rock specimens can also be directly employed in blasting experiments.

This approach enables the mechanical and physical properties of the model to more closely resemble those of natural rock. To this end, the advantages and disadvantages of both concrete models and in-situ rock specimens are summarized in Table 3.

**Table 3:** Advantages and disadvantages of model materials.

Model material	Advantages	Disadvantages
Concrete	Lower production cost, easy and rapid fabrication, and the possibility of embedding strain gauges within the specimen	Mechanical properties differ somewhat from in-situ rock
Natural Rock	Mechanical properties nearly identical to those of the field rock	Relatively higher cost, size is limited by cutting equipment and field sampling, and strain gauges cannot be embedded internally

**Fig. 10:**
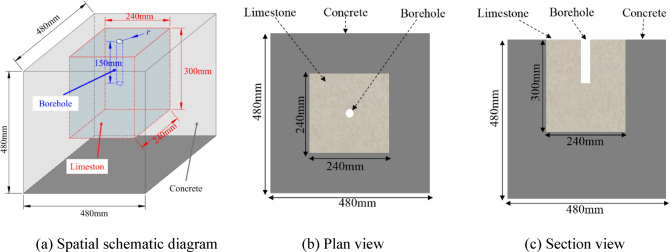
Blast physical modeling.

To better integrate the respective advantages of concrete materials and in-situ rock specimens, this study proposes an innovative method for constructing physical models. As illustrated in Fig. 10, the method involves using a “limestone core + concrete confinement” configuration. Specifically, limestone collected from the tunnel construction site is processed into rectangular specimens with dimensions of 240 mm × 240 mm × 300 mm to serve as the core blasting region. These limestone cores are then externally confined with high-strength concrete. This technique allows the material surrounding the cylindrical charge to better approximate natural rock properties, while the external concrete confinement effectively increases the overall model size and structural integrity.

**Fig. 11:**
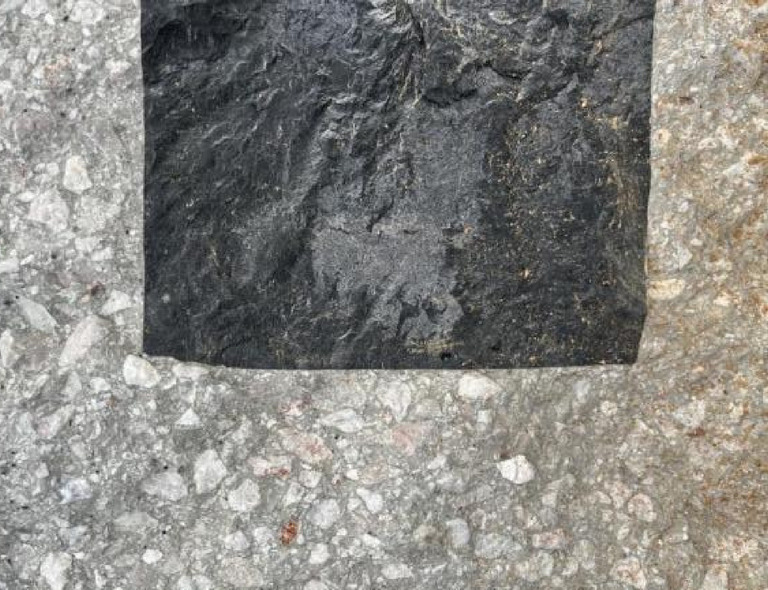
Internal section of the model.

In the field casting test, ordinary Portland cement was used as the cementing material, with a mix design comprising 380 kg/m^3^ of cement, 680 kg/m^3^ of sand, 1200 kg/m^3^ of gravel, and 170 kg/m^3^ of water, yielding a water-to-cement ratio of 0.45. A chemical admixture was added at 1–2% of the cement mass. This mix was used for casting the external concrete portion of the model. As shown in Fig. 11, after casting, the limestone cores and concrete demonstrated effective bonding, with no visible faults or cracks observed at the interface. All models were subjected to a curing period of 28 days prior to blasting tests.

**Fig. 12:**
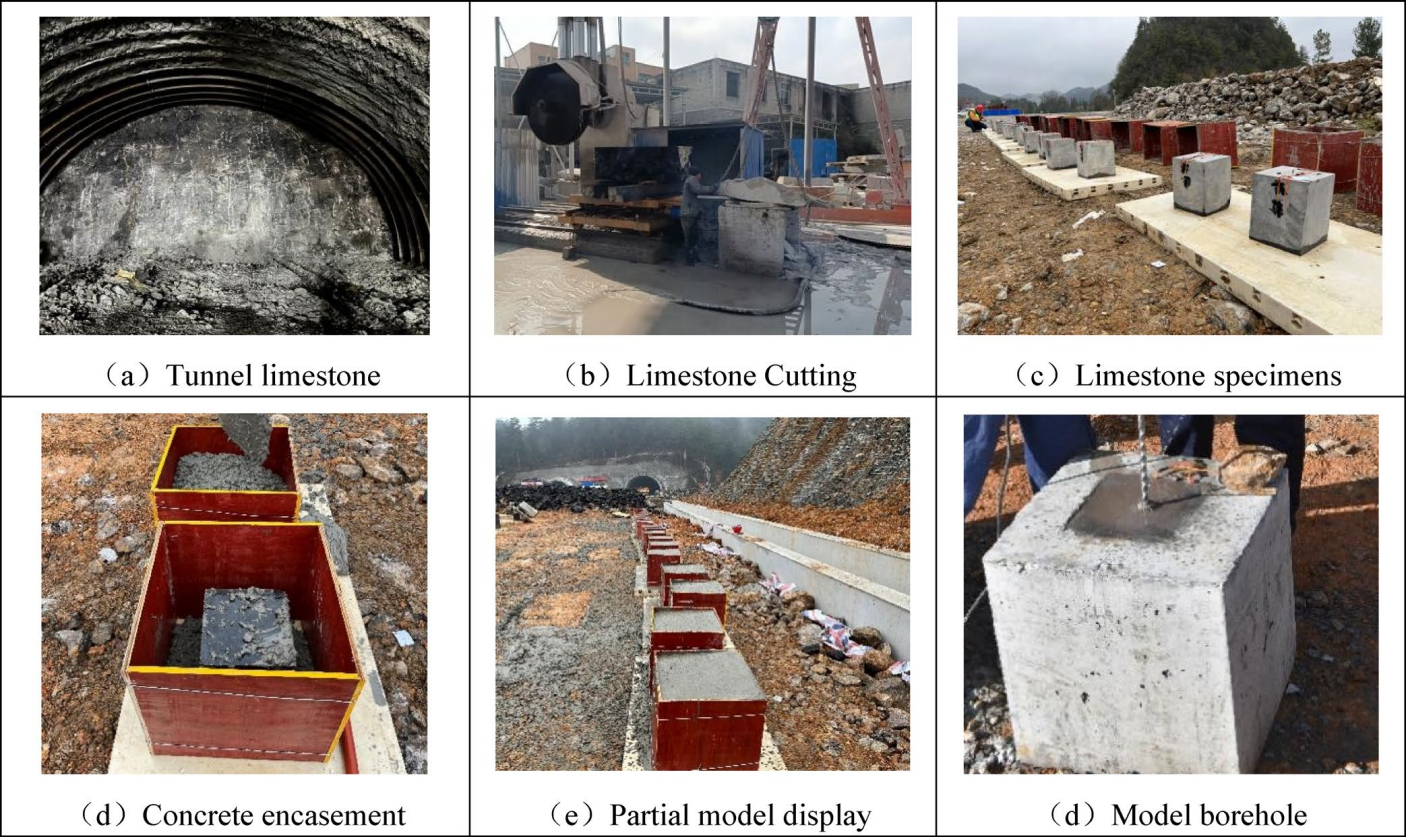
Blast physical model casting process.

To ensure the tests proceed smoothly, the test site was selected in a spacious, safe, and convenient roadbed near the tunnel construction site. The test area was leveled and secured with debris from tunnel blasting serving as additional confinement. Templates were fabricated according to the model dimensions, and a carbon steel base was used to ensure flatness (see Fig. 12).

**Fig. 13:**
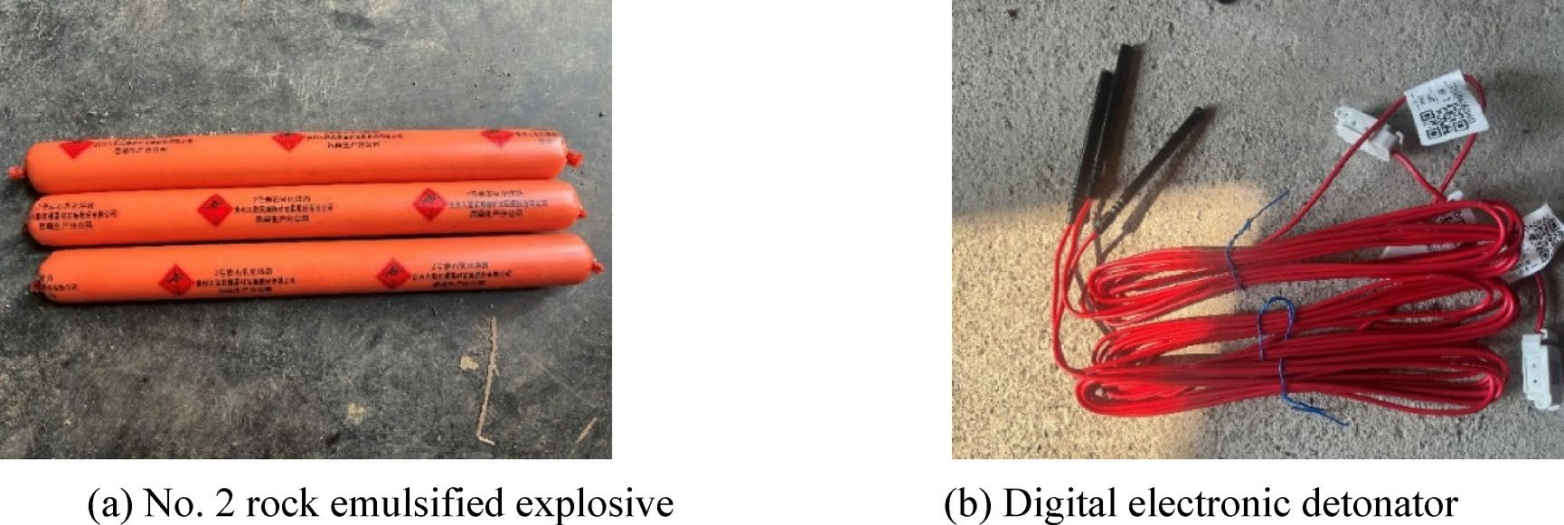
Blasting test explosives.

In addition, as shown in Fig. 13, this study proposes the use of No. 2 rock emulsion explosive—commonly employed in conventional tunnel blasting—as the blasting charge. This explosive has a density of approximately 1.0–1.2 g/cm^3^ and a detonation velocity of around 3500 m/s. Digital electronic detonators are used for initiation, with each detonator featuring an outer shell approximately 75 mm in length and 8 mm in diameter.

### Parameter design and implementation

**Fig. 14:**
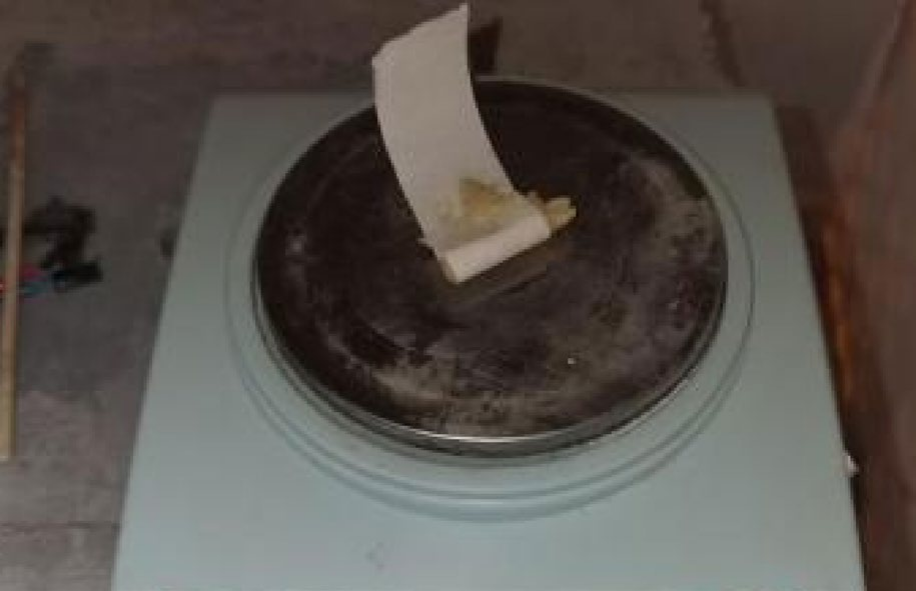
Explosive charge control.

**Fig. 15:**
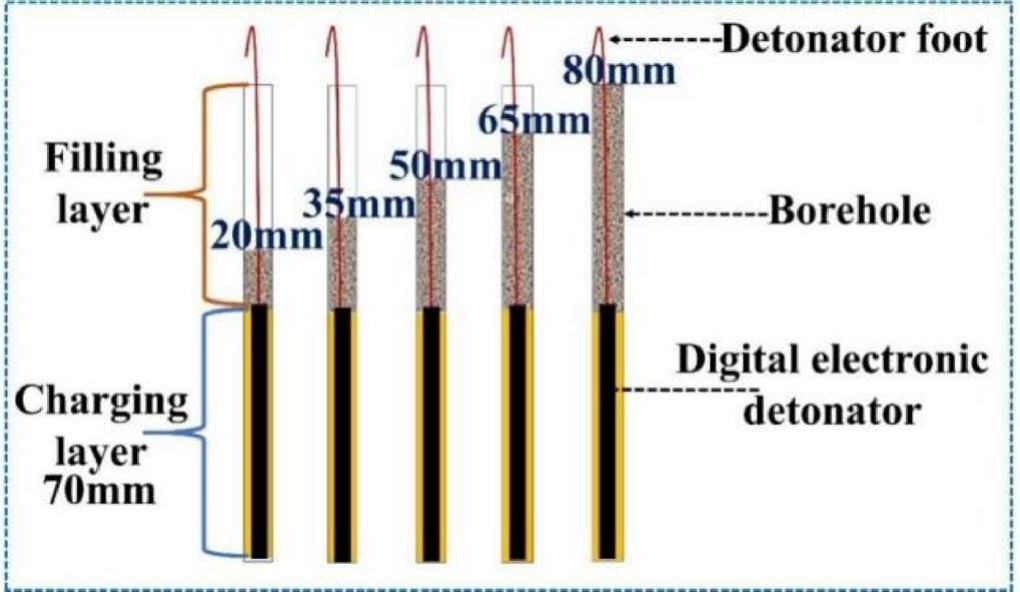
Schematic diagram of the charge structure under different experimental conditions.

Taking into account the physical dimensions of the digital electronic detonator, the borehole diameter is designed to be 10 mm, with a depth of 150 mm. The charge length is set to 70 mm, and the emulsion explosive is weighed using an electronic balance to ensure precise control of the charge mass for each test group, as illustrated in Fig. 14. The stemming lengths using rapid-setting gun mud are designed to be 20 mm, 35 mm, 50 mm, 65 mm, and 80 mm, respectively. The overall charging configuration is shown in Fig. 15.

## Analysis of model blasting test results

### Collection and statistics of blast fragmentation

**Fig. 16:**
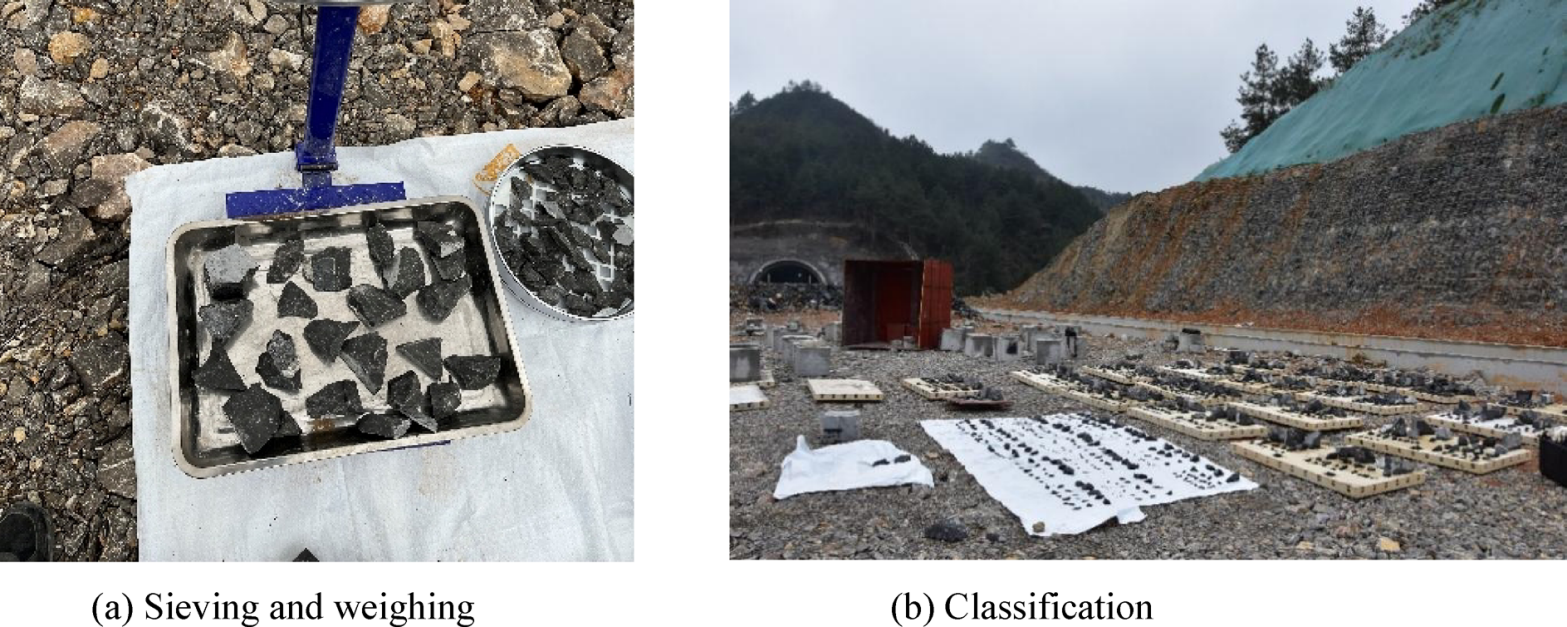
Field site for blast fragment collection and organization.

After each blasting test, the fragmented limestone pieces were individually collected and sorted. Based on the fragmentation distribution of each test group, the fragments were screened using square-mesh sieves of varying aperture sizes ranging from 20 to 60 mm. Larger rock blocks were measured directly using a steel ruler. Finally, all fragments were arranged in descending order of size, as shown in Fig. 16.

**Fig. 17:**
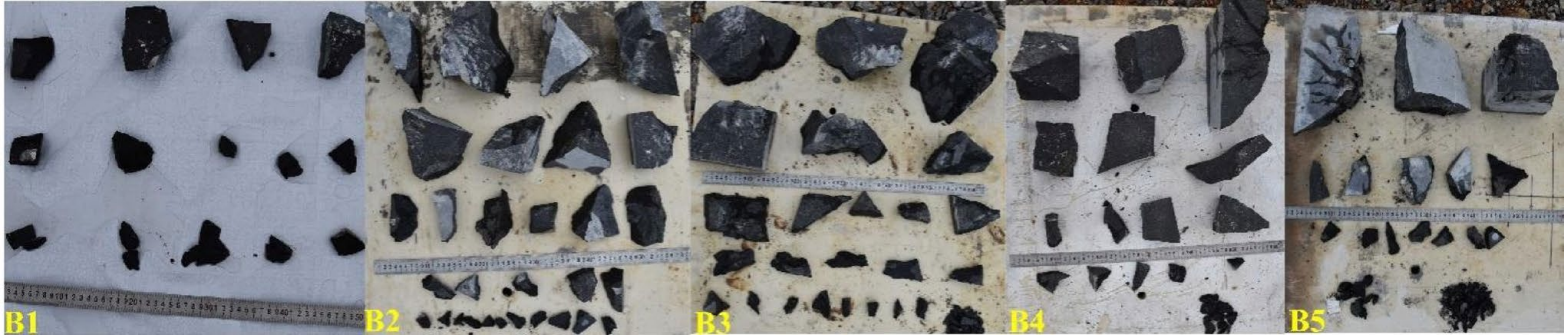
Blast block size for different filling conditions.

As shown in Fig. 17, we collected and organized the fragmentation data of limestone under different stemming lengths. Except for the small flyrock and fine particles that were not collected, the fragmentation data described below can be regarded as the total fragmentation of each test group.

It can be observed from Fig. 17 that the fragmentation characteristics of limestone vary significantly with different stemming lengths. When the stemming length is 20 mm, the number of fragments is relatively small, and the sizes are mainly concentrated in the fine fraction. As the stemming length increases, the number of fragments first increases and then decreases. When the stemming length reaches the maximum of 80 mm, the limestone primarily fractures radially along the borehole, forming three large blocks with poor uniformity.

According to the actual fragmentation results in each test group, we divided the particle size distribution into seven intervals: ≤ 20, 20–40, 40–60, 60–80, 80–100, 100–120, and 120–180 mm, denoted as i (i = 1–7). The corresponding statistical data are shown in Table 4. From Table 4, it can be seen that the total fragmentation mass was only 2.55 kg at a stemming length of 20 mm, which increased significantly to 35.17 kg at 35 mm (nearly 15 times higher). With further increases in stemming length, the total fragmentation mass tended to stabilize at around 35 kg.

**Table 4:** Blast block size screening statistics table.

Condition	Fragment size class/mm	≤ 20	20–40	40–60	60–80	80–100	100–120	120–180
B1	Mass/kg	0.38	0.63	0.49	1.05	0	0	0
	Cumulative mass/kg	0.38	1.01	1.50	2.55	2.55	2.55	2.55
	Mass percentage /%	14.90	24.71	19.22	41.18	0	0	0
	Cumulative mass percentage/%	14.90	39.61	58.82	100.00	100.00	100.00	100.00
B2	Mass/kg	3.11	6.79	8.65	10.49	6.13	0.00	0.00
	Cumulative mass/kg	3.11	9.90	18.55	29.04	35.17	35.17	35.17
	Mass percentage/%	8.84	19.31	24.59	29.83	17.43	0	0
	Cumulative mass percentage/%	8.84	28.15	52.74	82.57	100.00	100.00	100.00
B3	Mass/kg	2.98	4.15	2.13	5.51	8.32	7.38	5.14
	Cumulative mass/kg	2.98	7.13	9.26	14.77	23.09	30.47	35.61
	Mass percentage/%	8.37	11.65	5.98	15.47	23.36	20.72	14.43
	Cumulative mass percentage/%	8.37	20.02	26.00	41.48	64.84	85.57	100.00
B4	Mass/kg	0.98	1.68	1.64	1.36	3.69	5.94	20.84
	Cumulative mass/kg	0.98	2.66	4.30	5.66	9.35	15.29	36.13
	Mass percentage/%	2.71	4.65	4.54	3.76	10.21	16.44	57.68
	Cumulative mass percentage/%	2.71	7.36	11.90	15.67	25.88	42.32	100.00
B5	Mass/kg	0.33	1.21	0.65	1.35	0	0	33.62
	Cumulative mass/kg	0.33	1.54	2.19	3.54	3.54	3.54	37.16
	Mass percentage/%	0.89	3.26	1.75	3.63	0	0	90.47
	Cumulative mass percentage/%	0.89	4.14	5.89	9.53	9.53	9.53	100.00

**Fig. 18:**
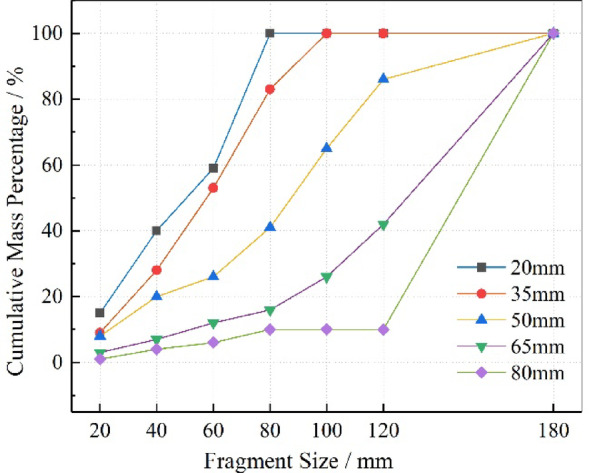
Blasting block size cumulative mass distribution curve.

From the fragmentation distribution curves (Fig. 18), it is observed that with increasing stemming length, the initial slope of the curve decreases, indicating a reduction in small fragments and an increase in large fragments. Excessive stemming length suppresses effective energy dissipation, leading to a higher proportion of large fragments.

### Quantitative analysis of blasting block size distribution characteristics

To more intuitively describe the effect of stemming length on fragmentation uniformity, the mean fragment size (*d*_*m*_) is introduced to represent the degree of rock fragmentation^49^:

34 $$d_{m} = \sum {(r_{i} d_{i} {)/}r_{i} }$$

where *d*_*i*_ is the average size of fragments in a given sieve interval, and *r*_*i*_ is the corresponding mass percentage of that interval.

Although the mean fragment size reflects the overall degree of fragmentation to some extent, it is insufficient as the sole criterion for evaluating blasting performance in engineering practice. This is because practical blasting requires not only sufficient fragmentation but also two key goals: (1) controlling the proportion of oversized blocks to ensure the continuity and efficiency of mucking, hauling, and mechanized operations, and (2) avoiding excessive fines, which cause energy waste and flyrock risks, while maximizing the range of effective fragment sizes for overall blasting optimization.

Based on this, combined with the actual fragmentation characteristics and model scale parameters, we defined fragments larger than 120 mm as the “oversized zone” and fragments smaller than 20 mm as the "over-crushing zone," in order to establish a more comprehensive evaluation system for blasting performance.

### Effect of stemming length on rock fragmentation characteristics

Finally, using the test data from Table 4 and Eq. (34), we calculated and analyzed key indicators such as mean fragment size, proportion of oversized blocks, and proportion of over-crushed fines under different stemming lengths. The results are shown in Table 5. As indicated, the minimum stemming length (20 mm) corresponded to the lowest mean fragment size of 49.39 mm, while the maximum stemming length (80 mm) increased it to 140.19 mm. Moreover, the proportion of oversized blocks was 0% at 20–35 mm, but reached a maximum of 90.47% at 80 mm. Meanwhile, the proportion of over-crushed fines remained relatively small, below 20%.

**Table 5:** Blast block size screening statistics table.

Condition	*δ*_*i*_/mm							*δ*/mm	Oversized zone /%	Over-crushing zone /%
	1	2	3	4	5	6	7			
B1-1	1.49	7.41	9.61	30.88	0	0	0	49.39	0	14.90
B1-2	0.88	5.79	12.30	20.88	15.69	0	0	55.54	0	8.84
B1-3	0.84	3.50	2.99	10.83	21.03	22.80	21.65	83.63	14.43	8.37
B1-4	0.27	1.39	2.27	2.63	9.19	18.08	86.52	120.37	57.68	2.71
B1-5	0.09	0.98	0.87	2.54	0	0	135.71	140.19	90.47	0.89

**Fig. 19:**
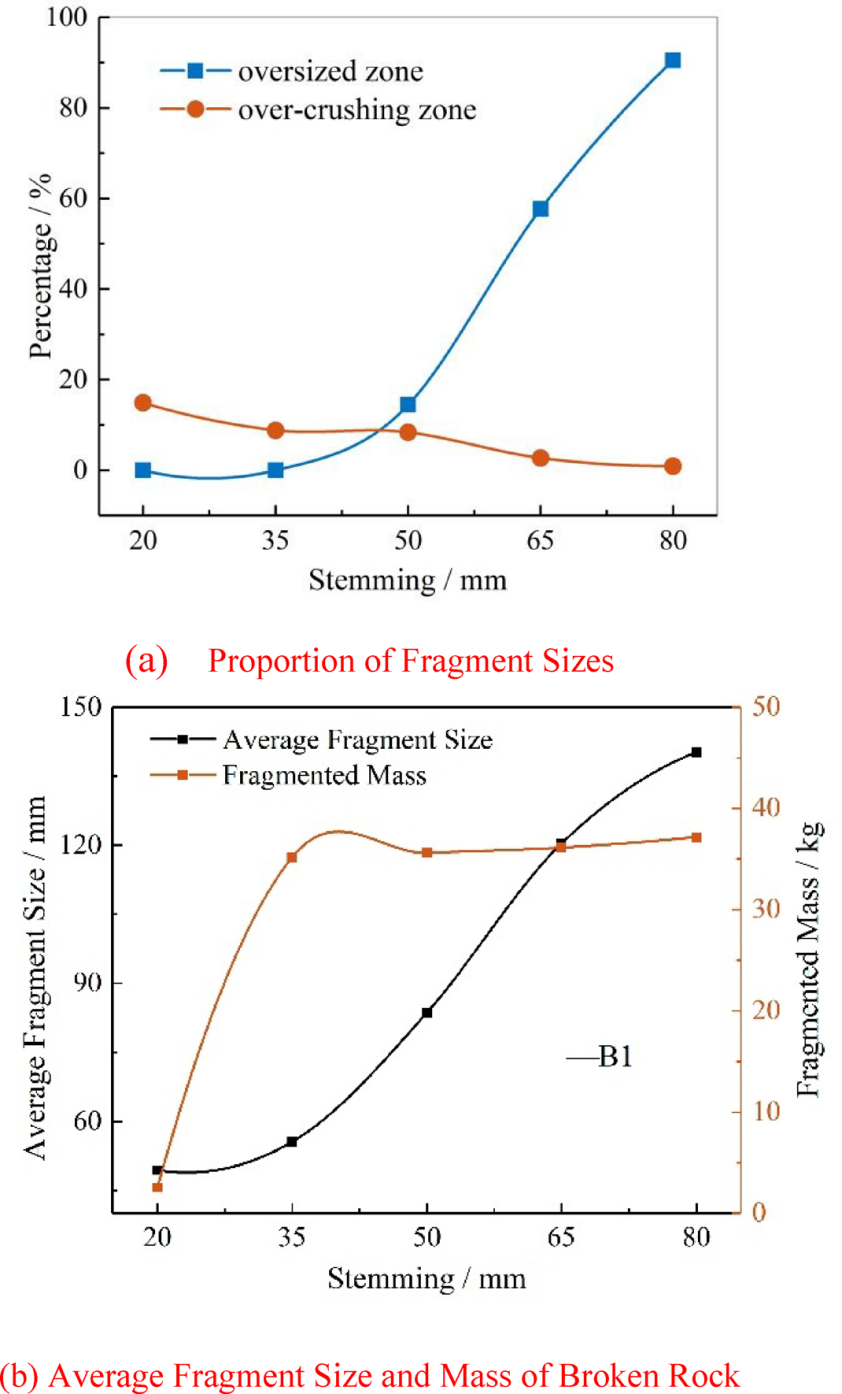
Blasting block size cumulative mass distribution curve.

As shown in Fig. 19, the proportion of oversized blocks and the mean fragment size followed a similar trend with stemming length. Within 50 mm, the initial slope of both indicators was small, indicating slow growth; beyond 50 mm, both increased rapidly. In contrast, the proportion of over-crushed fines decreased slowly and steadily with increasing stemming length. The total fragmentation mass increased rapidly at first and then stabilized, with the most significant changes occurring in the 20–35 mm range.

From the fragment photographs in Fig. 17, it can be seen that insufficient stemming length results in limited total fragmentation despite low mean fragment size and oversized proportion, indicating a lack of effective overall fragmentation. This suggests that mean fragment size and oversized proportion cannot serve as the sole indicators of blasting performance. Furthermore, when the stemming length is too long, energy release is restricted, leading to the formation of large blocks near the borehole collar. This increases both the mean fragment size and oversized proportion, thereby inhibiting fragmentation effectiveness. Overall, a stemming length of 50 mm was found to be optimal under the tested conditions.

### Analysis and verification of the optimal stemming length for the rapid-setting sealing material

To further validate the feasibility of the optimal stemming length model for the rapid-setting gun mud under multi-parameter coupling conditions, including:

1. **Rock Parameters.** Based on laboratory tests, the limestone has a longitudinal wave velocity $C_{P}$ = 2799 m/s. The Poisson’s ratio is derived from Eq. (12), and the Rayleigh wave velocity $C_{R}$ is taken as 0.87 times $C_{P}$, i.e., $C_{R}$ = 2435 m/s.
2. **Stemming Material Parameters.** According to laboratory test results, the density of the rapid-setting gun mud is 1600 kg/m^3^. $f$ conservative value, denoted as 0.055, is adopted for the sliding friction coefficient between the gun mud and the limestone.
3. **Explosive Parameters.** The explosive has a density of 1000 kg/m^3^ and a detonation velocity of $C = 4000$ m/s, the pressure $p_{0}$ can be calculated using Eq. (23).
4. **Drilling-Blasting Parameters.** Based on the model design, the borehole depth $l = 0.15$ m, and the charge length $l_{a} = 0.07$ m. The minimal resistance line $w = 0.235$ m, and the borehole diameter, which is equal to the charge diameter, $d = d_{a} = 0.01$ m.

In summary, by substituting the above parameters into Eq. (33), the following result is obtained:

35 $$l_{s2} = \frac{{(l_{a} d\rho_{0} - 2p_{x} t_{p}^{2} \lambda f - ld\rho_{0} ) + \sqrt {(l_{a} d\rho_{0} - 2p_{x} t_{p}^{2} \lambda f - ld\rho_{0} )^{2} - 2d\rho_{0} p_{x} t_{p}^{2} d} }}{{2kd\rho_{0} }}$$

36 $$l_{s2} = \frac{{(l_{a} d\rho_{0} - 2p_{x} t_{p}^{2} \lambda f - ld\rho_{0} ) - \sqrt {(l_{a} d\rho_{0} - 2p_{x} t_{p}^{2} \lambda f - ld\rho_{0} )^{2} - 2d\rho_{0} p_{x} t_{p}^{2} d} }}{{2kd\rho_{0} }}$$

37 $$l_{s1} = (53.26 \sim 72.63)\,\hbox{mm}$$

38 $$l_{s2} = (0.09 \sim 0.12)\,\hbox{mm}$$

In practical applications, stemming length cannot be less than 1 mm. Therefore, the optimal stemming length calculated by the model should be taken as 53.26 mm to 72.63 mm. In contrast, experimental results suggest that the optimal stemming length is approximately 50 mm, which is slightly lower than the theoretical value. This discrepancy mainly arises from the assumption of perfect coupling between the explosive and the borehole wall in the theoretical model, which is difficult to achieve in practice. Consequently, the theoretical charge mass is relatively larger, leading to an overestimation of stemming length. Nevertheless, the difference between experimental and theoretical values is only a few millimeters, demonstrating that the model predictions are consistent with actual blasting performance. The calculation methods and parameter values adopted in the model are reasonable and can effectively guide the conventional design of stemming length using rapid-setting gun mud. Future improvements may be achieved by incorporating large-scale field data for continuous refinement and optimization.

## Conclusions and discussion

1. The rapid-setting gun mud material proposed in this study exhibits excellent solidification properties and can quickly form an effective seal within the borehole. Its curing process relies primarily on hydration reactions, without releasing toxic by-products, ensuring both environmental friendliness and worker safety. These advantages provide a solid theoretical foundation for future engineering applications.
2. Model test results indicate that when stemming length is insufficient, both the average fragment size and the proportion of large fragments remain low, but the overall fragmentation mass is limited. Conversely, when stemming length is excessive, energy release is constrained, resulting in the formation of large rock blocks near the borehole collar, which increases both average fragment size and the proportion of large fragments. Therefore, optimizing stemming length is crucial for improving rock blasting performance, particularly by enhancing size distribution and increasing fragmented rock volume. Under the model test conditions, a stemming length of 50 mm is identified as optimal.
3. The proposed optimal stemming length model is in good agreement with experimental results. The assumptions and parameter selections involved are reasonable, demonstrating its applicability as a simplified algorithm for conventional borehole stemming design. The model for optimal stemming length is expressed as follows:
  $$l_{s} = \frac{{(l_{a} d\rho_{0} - 2p_{x} t_{p}^{2} \lambda f - ld\rho_{0} ) + \sqrt {(l_{a} d\rho_{0} - 2p_{x} t_{p}^{2} \lambda f - ld\rho_{0} )^{2} - 2d\rho_{0} p_{x} t_{p}^{2} d} }}{{2kd\rho_{0} }}.$$
4. Since this study is still in the preliminary exploratory phase and the sample size is limited, there are certain limitations. Future research will focus on large-scale field blasting application experiments for further validation. Additionally, we plan to explore the design of a mechanized filling operation vehicle, which will use a mechanical pumping method to inject A and B slurry mixtures into tunnel boreholes, gradually realizing the mechanized filling of tunnel boreholes.
5. In addition, this study primarily focuses on model experiments and has not yet considered the impact of external environmental variations at actual tunnel blasting sites on the application of rapid-setting stemming material. Factors such as tunnel site temperature, humidity, burial depth, and surrounding rock fragmentation may all influence the effectiveness of the rapid-setting stemming. Future research can focus on the variation patterns of these parameters, which represents a research direction with significant potential.

## Data Availability

All data generated or analysed during this study are included in this published article.
